# How participation in health promotion affects peer-experts and experts-by-experience from vulnerable neighborhoods: a case study from The Netherlands

**DOI:** 10.1093/heapro/daag035

**Published:** 2026-03-18

**Authors:** Samantha Elkhuizen, Bente van Aken, Emma Stulen, Dagmar Niewold, Annemarie Wagemakers, Kristina Thompson

**Affiliations:** Health and Society Chair Group, Department of Social Sciences, Wageningen University & Research, PO Box 8130, 6700 EW Wageningen, The Netherlands; Health and Society Chair Group, Department of Social Sciences, Wageningen University & Research, PO Box 8130, 6700 EW Wageningen, The Netherlands; Health and Society Chair Group, Department of Social Sciences, Wageningen University & Research, PO Box 8130, 6700 EW Wageningen, The Netherlands; Certified Expert-by-Experience in Poverty & Health, Amersfoort, The Netherlands; Health and Society Chair Group, Department of Social Sciences, Wageningen University & Research, PO Box 8130, 6700 EW Wageningen, The Netherlands; Health and Society Chair Group, Department of Social Sciences, Wageningen University & Research, PO Box 8130, 6700 EW Wageningen, The Netherlands

**Keywords:** peer-experts, peer support, experiential knowledge, health promotion, social domain, health inequalities, health and care workforce

## Abstract

Residents of vulnerable neighborhoods often experience economic hardship, poor health, and social exclusion. Health promotion in vulnerable neighborhoods is often unsuccessful due to residents’ mistrust toward professionals and governmental institutions and misaligned interventions. Engaging experts-by-experience and peer-experts, people drawing on lived experience to support others, offers a promising means to overcome these hurdles. While this approach is gaining ground in the social domain, little is known about its impact on the experts themselves. Therefore, this study addresses the question: “What is the impact of being an expert-by-experience or peer-expert within the social domain on the expert-by-experience and peer-experts themselves?” Using Participatory Action Research, we conducted seventeen participatory observations and nine semi-structured interviews with two peer-experts, one expert-by-experience, and nine professionals in a Dutch city. A reflexive thematic analysis identified four impacts of the expert-by-experience and peer-expert role: (1) feeling connected—through mutual recognition and community engagement; (2) feeling valued—by contributing meaningfully and being respected; (3) personal growth—gaining self-reflection, self-acceptance, and self-confidence; and (4) acquiring transferable skills and future prospects—such as language skills, boundary-setting, and employment opportunities. Challenges included balancing pressures associated with role identity, financial insecurity, and boundary-setting. We conclude that, working as an expert-by-experience and peer-expert offers personal benefits, but also presents risks without adequate support. Reciprocal learning between peer-experts, as well as supportive environments, and focus on financial security are needed to ensure sustainability. Ultimately, integrating experiential knowledge may help health promotion initiatives building trust, address health inequities, and achieve lasting impact in vulnerable neighborhoods.

Contribution to Health Promotion

- Being an expert-by-experience or peer-expert boosts confidence, self-esteem, provides a sense of purpose through meaningful contribution based on lived experience, and opens pathways to education or employment.
- When integrating experiential knowledge into health promotion initiatives, the following challenges for experts-by-experience and peer-experts should be considered: difficulties in setting boundaries, which can affect work-life balance; mistrust and stress regarding financial compensation; and resurfacing of painful memories.
- To foster positive impacts and protect the well-being of experts-by-experience and peer-experts, supportive supervision, reciprocal learning, and organizational recognition are required to provide guidance, clear role descriptions, and opportunities for development.

## Background

Residents in vulnerable neighborhoods—areas where socioeconomic disadvantage, limited access to resources, and environmental stressors are concentrated—are among the most likely in society to encounter economic hardships, poorer health, social isolation, and crime (Cubbin *et al.* 2008, Robinette *et al.* 2017, Kullberg *et al.* 2021). They often face multiple, overlapping challenges across areas of their lives, such as difficulties with parenting, poor living conditions, poor health, unemployment, poverty, and psychosocial well-being (Abidi *et al.* 2018, Evenboer *et al.* 2018, Verloo and Ferier 2021). Health promotion programs that address these broader determinants of health could be beneficial; yet, professionals often find it difficult to reach and engage residents in vulnerable neighborhoods consistently (Sokol and Fisher 2016). A barrier is the widespread mistrust toward health professionals and governmental institutions, which discourages residents from seeking or accepting support (Verloo and Ferier 2021). Also, policies and programs frequently fail to align with the lived realities of residents in vulnerable neighborhoods (Van der Kooij and Keuzenkamp 2018).

A promising solution for better aligning health promotion programs with the needs of residents of vulnerable neighborhoods is engaging experts-by-experience or peer-experts in the social domain. Experts-by-experience and peer-experts are individuals with firsthand experiences with limitation, exclusion, disruption, and/or other radical life-determining circumstances. They share this knowledge with others or use it to benefit others (Van der Kooij and Keuzenkamp 2018). We define experts-by-experience as individuals who have transformed their lived experience into certified expertise through formal education, and who are formally involved in sharing that knowledge professionally. We use the term peer-experts to refer to individuals, often citizens who reside in the neighborhood, who have received training and share their personal or experiential knowledge about living in a vulnerable position with others or use it to benefit others nonprofessionally. While experts-by-experience and peer-experts have been widely integrated into mental health services since the 1990s (Shalaby and Agyapong 2020), their role in the social domain, particularly for health promotion, has only emerged more recently (Van der Kooij and Keuzenkamp 2018, Keuzenkamp and Van Hoorn 2022).

The role of experts-by-experience and peer-experts differs considerably between mental health care and the social domain. In mental health care, their focus is on recovery support through individual guidance, group work, or contributing to treatment, within often formalized roles that include training and supervision (Jacobson *et al.* 2012, King and Simmons 2018). In the social domain, by contrast, experts-by-experience and peer-experts may address a wider range of issues, such as poverty, domestic violence, and social exclusion. Experts-by-experience contribute to the development of policy and health promotion programs, or act as speakers raising awareness about particular topics. Peer-experts act as community connectors, offering support and bridging gaps between residents and institutions, often in informal roles without standardized job descriptions or employment conditions. Their work is more context-dependent and varies by setting and target group. While activities across both sectors may overlap, such as sharing lived experiences, offering social support, providing advocacy, facilitating access to resources, or contributing to education, our study focuses specifically on the unique role and impact of experts-by-experience and peer-experts in the social domain.

Previous research suggests that the work of experts-by-experience and peer-experts may positively impact clients and residents, with reported outcomes including increased confidence, resilience, and self-esteem; improved self-management; an expanded social network; a sense of understanding and acceptance; and reduced stigma (Repper and Carter 2011, King and Simmons 2018, Van der Kooij and Keuzenkamp 2018, Shalaby and Agyapong 2020). Furthermore, involving experts-by-experience and peer-experts in the design process of interventions has been associated with improved acceptability and effectiveness (Schilling *et al.* 2021). However, evidence remains context-dependent, and questions persist regarding the mechanisms, sustainability, and broader added value of experiential knowledge. Moreover, while these studies highlight the positive impact experts-by-experience and peer-experts can have on others, less is known about how taking on this role affects the experts themselves.

A few studies in the mental health field have explored the impact that working on the basis of experiential knowledge has on experts-by-experience and peer-experts, showing that they feel valued and respected, with the role contributing to increased self-esteem and confidence (Repper and Carter 2011, Gillard *et al.* 2022, Happell *et al.* 2022). The work also contributes to personal recovery, a sense of belonging, greater meaning in life, and access to educational and financial opportunities (Gillard *et al.* 2022). At the same time, negative effects have been reported, like stress, pressure to succeed as pioneers, identity struggles, team tensions, and difficulties in setting boundaries (Repper and Carter 2011, Gillard *et al.* 2022). However, these studies have been conducted in the mental health domain, where, as outlined above, the context and roles of experts-by-experience and peer-experts differ significantly from those in the social domain.

This study therefore addresses the following research question: “What is the impact of being an expert-by-experience or peer-expert within the social domain on the expert-by-experience and peer-experts themselves?” Our aim is to deepen the understanding of how working on the basis of experiential knowledge impacts the individuals involved, thereby informing strategies to strengthen the position of experts-by-experience and peer-experts as well as improving their support in practice.

## Methods

### Study design and ethics

An explorative qualitative research design was used to investigate the impact of being an expert-by-experience or peer-expert within the social domain. The study followed a Participatory Action Research (PAR) approach, in which the experts and professionals were actively involved in co-creating the research process. PAR is aimed at both facilitating change in practice and evaluating that practice through iterative cycles of planning, action, observation, and reflection (Zuber-Skerritt 2001, Baum *et al.* 2006). The research question in this study was a local request from a youth work organization within the context of a broader study addressing health inequities (Wagemakers *et al.* 2025). In this study, we followed the implementation of working with experiential expertise. Two PAR cycles were conducted: the first aimed to explore the roles of peer-experts and experts-by-experience and to identify good practices from other contexts, and the second focused on examining the impact of these roles on the experts themselves. Meetings with researchers and local stakeholders were held every 6 weeks, and more frequently during data collection. Data collection methods were developed in close collaboration with participants and tailored to their needs and preferences. Insights gained during the research were continuously used to further shape and refine the study design and research questions. This study was conducted from September 2022 to June 2024. Ethical approval for this study was granted by the Research Ethics Committee of Wageningen University & Research (approval numbers: 2022-153-Elkhuizen; 2023-102).

### Setting and study population

This study took place in a large Dutch municipality (∼230 000 inhabitants), where peer-experts are active in three neighborhoods designated as vulnerable. These neighborhoods face significantly lower quality of life than the city as a whole, with disproportionately high social and health disadvantages. Many households experience unemployment, debt, poor living conditions, health problems, discrimination, and crime. Moreover, many residents have a migration background. Countries of origin include, amongst others, Morocco, Turkey, Syria, Somalia, Ethiopia, Poland, and Venezuela. Local health promotion initiatives have been launched in each neighborhood to promote equal opportunities for children's development and health. A key component of these initiatives is integrating experiential knowledge. Peer-experts and experts-by-experience contribute to identifying and addressing underlying determinants of health, such as education and early childhood development, language proficiency, access to supportive services, social inclusion, and socioeconomic conditions. This study includes three types of peer-experts active in the municipality (referred to as A, B, C), and one expert-by-experience (Table 1). All of the municipality's peer-experts are female.

**Table 1:** Characteristics of the expert-by-experience and peer-experts included in this study.

Title	Target audience	Main tasks	Number of experts	Since	Support	Training	Reward
Peer-experts A	Parents of children aged 2.5–4 years or in the first 2 years of primary education with a migration background living in the north of the municipality	Supporting parents with children at risk of (language) disadvantage in accessing additional education	10	2019	Supervision by social work professionals	Five training sessions covering topics such as communication skills, self-reflection, and the Dutch education system	€10 (cash or gift card) per 30-minute session
Peer-experts B	Parents of children transitioning from primary to secondary education with a migration background living in the north of the municipality	Supporting parents during the school transition phase	8	2023	Supervision by social work professionals	Five training sessions covering topics such as communication skills, self-reflection, and the Dutch education system	€10 (cash or gift card) per 30-minute session
Peer-experts C	Parents with young children living in the west and south of the municipality	Supporting local families and translating their needs to professionals	4	2022	Supervision by youth workers	Tailored training program	€10 gift card per hour (approximately 4 hours of work per week)
Expert-by-experience	Children, youth and parents living in the west of the municipality	Connecting, motivating, and supporting children, youth, and parents; advocating for experiential knowledge	1	2012	Not applicable	Tailored training program by the employing organization and education program offered by a recognized experiential expertise organization	Salary

Ten peer-experts A inform and guide parents of young children at risk of (language) disadvantage toward additional education. Eight peer-experts B support parents (with limited Dutch language skills) whose children are transitioning from primary to secondary education. Four peer-experts C are active in two neighborhoods and support parents with a variety of issues such as unemployment, poverty, and children’s development, and translate the needs of residents to professionals. The expert-by-experience is employed by the municipal youth work organization and is responsible for connecting with, motivating, and supporting children, youth, and their parents. She also serves as a pioneer in integrating experiential knowledge within her organization.

### Positionality of the researchers

The research team consisted of S.E., B.v.A., and E.S., all three white, cisgender women with an MSc degree. None of the researchers grew up in a vulnerable neighborhood, although they share strong social justice values and a professional background in public health, with expertise in community-based health promotion and participation. While some had prior experience working with local communities, none had previously collaborated with peer-experts or experts-by-experience. The team was aware of the potential power dynamics inherent in engaging with the experts and professionals and actively reflected on their own assumptions throughout the research process. Data collection was carried out in close collaboration with professionals who maintained strong ties with the peer-experts, helping to build trust and rapport. Reflexivity was fostered through regular team discussions, joint data analysis, and support from an experienced researcher specialized in community health promotion and stakeholder inclusion (A.W.), a certified expert-by-experience in poverty and health (D.N.), and a researcher specialized in health inequalities (K.T.).

### Data collection

Data collection took place from September 2022 to December 2022 and from November 2023 to March 2024. It consisted of participatory observations and interviews (Table 2). The researchers (B.v.A., E.S.) were invited by the project leader of the municipal youth work organization and group facilitators to become part of the groups of peer-experts and attend meetings and activities at which peer-experts were present. Their participation was intended to build trust and promote mutual learning, while minimizing the burden on peer-experts by ensuring that they did not need to participate in research activities. To further safeguard this, only one researcher could attend meetings at a time.

**Table 2:** Overview of the data collection.

Method of data collection	Number of interviews and observations	Specifications		Duration	Description
**Interviews**	9 (12 participants)	Expert-by-experience	1 (1 participant)	50–77 minutes (average 58 minutes)	Semi-structured interviews exploring experiences, motivations, roles, and impact of the work.
		Peer-experts A & B	1 (2 participants)		
		Professionals	7 (9 participants)		
**Participatory observations**	17	Peer-experts A & B	3 Peer-experts group meetings (6–12 participants; 2–7 professionals, 4–5 peer-experts)	1 hour	Monthly get-togethers to discuss ongoing issues, share experiences, and reflect on questions.
			1 Meeting at the parent meeting place (∼25 participants)	1.5 hours	Place at an elementary school where a diverse range of activities is offered to parents during school hours, such as Dutch or math classes, coffee mornings, walk-in consultation hours, and presentations. Several peer-experts are present.
			1 Informal conversation (4 participants; 2 professionals, 2 peer-experts)	45 minutes	In-depth discussion about the lives and work of peer-experts.
			2 Co-design sessions (∼30 participants; half professionals, half peer-experts)	2–3 hours	Development of interventions for a neighborhood program about the first 1000 days of children; interactive and creative methods were used to identify challenges and solutions.
		Peer-experts C	5 Peer-experts group meetings (5–8 participants; 2–4 professionals, 3–4 peer-experts)	1–2 hours	Monthly get-togethers to discuss ongoing issues, share experiences, and reflect on questions.
			2 Project team meetings (12–13 participants)	2 hours	Monthly meetings discussing progress of the program, of which peer-experts are part.
			1 Working group peer-experts (3 participants)	1.5 hours	Presenting knowledge about peer-experts from the literature and this study.
			2 Annual project days (∼30 participants)	2.5 hours	Annual project day where program progress is presented to partners from various organizations. Includes participatory workshops, such as a presentation on the work of peer-experts, and a Q&A session to discuss their experiences and insights.

Participatory observations were conducted over seventeen meetings (Table 2). The researchers (B.v.A., E.S.) immersed themselves in the groups of peer-experts, both observing and engaging with participants while collecting data. This approach provided firsthand insights into the group’s culture, behaviors, and social dynamics (Guest *et al.* 2013). Observations took place in meetings at various settings, including the group meetings of the three types of peer-experts, a gathering at a meeting place for parents where peer-experts were present, an informal conversation with peer-experts, co-design sessions of a new intervention in the neighborhood with peer-experts and professionals, project-team meetings, a meeting of the working group on peer-experts, and the annual project day of the program in which peer-experts C participate. Detailed notes were taken during all sessions, keeping the topics from the interview guide in mind.

An interview guide aligned with the project’s goals was developed in co-creation with the project team and checked by a certified expert-by-experience (D.N.) (Supplementary File S1). In the interview guide, we used the principles of Appreciative Inquiry, which makes use of storytelling (Bäckström *et al.* 2018, Frankel *et al.* 2020). Questions were formulated using three of the four Ds of Appreciative Inquiry: Discovery, Dream, and Design. “Discovery” focused on tasks and experiences as expert-by-experience or peer-expert, and the training and support received. “Dream” encouraged participants to envision the ideal future as expert-by-experience or peer-expert. “Design” focused on what would be needed to move toward these envisioned futures.

Using this interview guide, nine in-depth, semistructured interviews were conducted with twelve participants, including one expert-by-experience, two peer-experts and nine professionals (Table 2). The professionals interviewed were either guiding the peer-experts or closely related. They were a project leader and a family support worker from the municipal youth work organization, an educational worker from a high school, coordinators of the parent room, municipal staff, and members of the project team. In consultation with supporting professionals, we focused on interviewing professionals rather than peer-experts to avoid overburdening peer-experts, respect the trust-based relationships involved, and draw on professionals’ close collaboration with peer-experts to reflect on their roles, contributions, and successes and challenges within the neighborhood. The interviews were audio-recorded to allow for verbatim transcription following verbal (*n* = 7) or written (*n* = 5) consent. Participants provided verbal or written consent after receiving study information and answering four informed-consent questions, all of which had to be answered affirmatively to participate. The interviews lasted on average 58 minutes (ranging from 50 to 77 minutes), and were conducted by two researchers (S.E. and B.v.A./E.S.), either online or at a location chosen by the participants.

Findings were fed back to the project by developing factsheets that summarized the results and by participating in a working group where broader knowledge about peer-experts was shared. These contributions informed the development of a profile for peer-experts and generated insights into the impact of their work, as well as the need for support, training, education, and further professional development, including the continued evolution of the role itself.

### Data analysis

The transcripts of the interviews and meeting notes were analyzed using a reflexive thematic analysis which focuses on identifying, analyzing, and reporting patterns within data (Braun and Clarke 2006, 2019). Transcripts were loaded in Atlas.ti qualitative data analysis software and the analysis was performed in six phases.

In phase 1, two researchers (S.E. and B.v.A./E.S.) read the transcripts and notes to become immersed in and familiarized with the data. In phase 2, inductive coding was conducted using both semantic and latent approaches. Semantic codes captured explicit and surface-level meanings, while latent codes identified more interpretative, implicit meanings (Braun and Clarke 2006). Four interviews were double-coded by two researchers (S.E. and B.v.A./E.S.) to develop a shared understanding and reach consensus on the code list. In phase 3, codes were clustered into overarching themes and subthemes. Initial themes described in a MSc thesis were: the character of the expert-by-experience and peer-experts, the role of the expert-by-experience and peer-experts described in terms of four forms of social support (informational, instrumental, emotional, and appraisal), positive impacts of being an expert-by-experience or peer-expert which were feelings of fulfillment, cultivating personal growth, and acquiring skills and future prospects, negative impacts of being an expert-by-experience or peer-expert, and the need for support. For this paper, the codes were intertwined into three overarching themes, each consisting of several subthemes: motivations, roles, and impacts (Supplementary File S2). In phase 4, themes were reviewed to ensure an accurate representation of the data set. In phase 5, themes were further defined, and clear definitions and names were developed. In phase 6, findings were synthesized through a narrative summary, supported by illustrative quotes. This paper focuses primarily on the impacts of the work on the expert-by-experience and peer-experts, but because these impacts are closely intertwined with the reasons why the expert-by-experience and peer-experts engage and how they take on their roles, we first outline their motivations and roles. In phases 3–6, all researchers discussed codes and themes and reached consensus.

## Results

### Motivations of the expert-by-experience and peer-experts

The primary motivation for the expert-by-experience and peer-experts to engage in this role is their intrinsic desire to help others. Many of them express a deep commitment to improving the neighborhood and supporting people in vulnerable situations. Their work is often driven by a sense of social responsibility and empathy rooted in their own lived experiences.

An important motivation is the ambition to address issues they personally recognize in their community. One peer-expert stated: “I see so many lonely women, I want to do something for them.” (meeting peer-experts C). Several participants also mentioned improving health and prospects for the next generation to create equal opportunities for every child in the neighborhood.

Another motivation is the social cohesion and mutual respect between residents of different cultural backgrounds. Many experts wish to foster more connection and understanding within their culturally diverse neighborhoods. As one peer-expert explained, they aim to create a shared sense of belonging in the neighborhood. This includes promoting mutual respect and encouraging people to be open to others’ perspectives and traditions.

While intrinsic motivation clearly dominates, factors such as personal development and, to a lesser extent, financial incentives also provided motivation. However, these were typically seen as secondary to the desire to make a meaningful contribution to the community.

### The role of the expert-by-experience and peer-experts

Although the expert-by-experience and peer-experts took on diverse roles, common activities included mentoring and skill-building, connecting to resources, community building, and advocacy.

#### Mentoring and skill-building

A core part of the work of the expert-by-experience and peer-experts involves providing (in)formal mentorship and guidance. In this study, peer-experts are part of the communities they work with. They share experiences with residents or have the same cultural and linguistic background. This enables them to connect quickly and on an equal footing. Their ability to “speak the same language,” both literally and figuratively, helps them to build trust and provide culturally appropriate support. As one professional noted:

“They [peer-experts] live in the neighborhood themselves, are in the community themselves. Yes, which also creates trust or a bond with each other. I think a professional needs to have three conversations before trust is established. And such a peer-expert might reach a point in the first conversation that a professional cannot yet reach.” (I007, professional).

Mentoring involves providing information on practical matters, such as navigating the Dutch school system, assisting with official correspondence, and accompanying residents to school meetings or medical appointments. Peer-experts also support parents by providing advice, listening, offering emotional support, or giving a hug.

Additionally, the expert-by-experience and peer-experts help others recognize their own strengths and grow personally. Many act as role models, demonstrating that change and achievement are within reach and inspiring others to envision new opportunities for themselves:

“These women tell themselves the story of: ‘I'm a single mother, so that's not for me.’ … And then a woman like that comes along, a Moroccan woman, who just nails that training and gets a permanent contract at school. And then those women think, ‘Oh!’ And I think that is what such an ambassador brings to the neighborhood. That other women look at her and think: ‘Oh, but if she can do it[, so can I].’’ (I008, professional).

#### Connecting to resources

The expert-by-experience and peer-experts are connecting residents to additional help and services. Because of their relatability, or neighborly position, their support feels safer and more approachable. For example, one peer-expert was able to discreetly refer a child to the school breakfast program: “Because of her position, it is accepted when she refers people to, e.g. the school breakfast. She sees who could use a sandwich. For a professional, that would be way too tense to say and would hardly be accepted.” (meeting peer-experts C). Some peer-experts offer direct practical support, such as donating groceries or contributing to school expenses, from their own resources or through their informal or formal networks.

#### Community building

The expert-by-experience and peer-experts play a vital role in fostering social cohesion within their multicultural neighborhoods, which include over 120 nationalities. They organize and host various activities, such as communal dinners, tea mornings, or events at the toy library, which creates spaces where residents can meet, connect, and build trust across cultural boundaries. Additionally, experts lower the threshold for engagement in existing neighborhood initiatives.

#### Advocacy

Finally, the expert-by-experience and peer-experts act as advocates within their communities and toward professionals and institutions, promoting the value of experiential knowledge and representing the needs and perspectives of residents. Their close contact with residents allows them to pick up on emerging issues, needs, and concerns. As one professional put it: “They feel what is happening, they see what happens on the street, they hear things from their own children. And I think that that makes their position very powerful.” (I003, professional).

Peer-experts C represent families in their neighborhood through their shared lived experiences and translate this for professionals into concrete suggestions for improvements in practice or policy, such as contributing to agenda-setting of local programs, co-developing of new initiatives, and advising on better alignment of multidisciplinary meetings with residents’ lived realities. The expert-by-experience is a speaker at conferences to raise awareness on the value of lived experience in organizational contexts.

### The impact of working as an expert-by-experience and peer-expert

Working as an expert-by-experience or peer-expert has a profound personal impact. We identified four ways in which this was experienced, both positively and negatively, namely: feeling connected, feeling valued, cultivating personal growth, and acquiring transferable skills and future prospects.

#### Feeling connected

Meeting other people with similar experiences creates a feeling of connectedness amongst the expert-by-experience and peer-experts. However, it might also resurface personal experiences, cause frustration, and challenge work-life balance.

The role of the expert-by-experience and peer-experts fosters a strong sense of connectedness with the broader community, fellow experts, and professionals. The expert-by-experience and peer-experts recognize themselves in the people they support, which motivates them to help. One professional illustrated this: “Because [peer-expert] says: ‘I came here in the same way. And I know exactly how they feel.’ So, she really understands that very well. That’s also a motivation to help others.” (I012, professional). Additionally, peer-experts described how meeting other peer-experts with similar backgrounds in training sessions or regular meetings creates a powerful sense of mutual recognition and solidarity. Sharing personal stories allows them to identify with one another, which in turn strengthens group cohesion. As one professional described: “The best part of the training was that everyone spoke a different language and had a different culture, but ultimately we all spoke the same language [figuratively referring to mutual understanding].” (meeting peer-experts B). Being part of a group of peer-experts contributes to feelings of pride and collective purpose. These groups often develop their own support networks, sparring over challenges, sharing strengths, and dividing responsibilities based on their personal expertise. Additionally, peer-experts often have a warm and close relationship with the professionals guiding them. The peer-experts highly value the support and understanding they receive from their environment, organization or professionals.

However, this feeling of connectedness also has downsides. The expert-by-experience and peer-experts regularly encounter residents whose struggles closely mirror their own past or present situations. This may also resurface painful memories or evoke frustration, even when being trained for these situations, particularly when no solutions can be offered. Interactions with institutions like the municipality can add to this frustration when peer-experts feel their perspectives are not acknowledged. As was mentioned by a peer-expert: “We still have a lot of work to do. […] Like: ‘Hey, take our culture into account. Take our religion into account. Because things are not the same for us.’ […] You have to talk in easy language in the neighborhood. But I have to talk in municipal language there. So sometimes I really feel like… [sighs].” (I014, peer-expert).

The expert-by-experience describes that sharing personal experiences can feel vulnerable. The (lived) experiences are her work, and cannot be hidden when having a bad day. As the expert-by-experience put it: “I always say: you're walking around naked.” (I009, expert-by-experience). The expert-by-experience and peer-experts find it difficult to disconnect from their role, often leading to stress. Being both a community member and peer-expert blurs the boundaries between work and personal life. The professionals supervising peer-experts recognize this tension and try to offer support, encouraging them to set boundaries, respect their own limits, and take care of themselves.

#### Feeling valued

The expert-by-experience and peer-experts feel valuable by the sense of making a difference, and the positive feedback and remuneration they receive. However, feeling valuable can be threatened by challenges related to receiving financial compensation when experts also receive social security benefits, as well as by the pressures associated with their role identity.

All experts described a sense of fulfillment from the feeling that they are making a meaningful difference. For instance, a professional shared about a peer-expert: “She [a peer-expert] had spoken to the mother so convincingly, and spent almost half an hour telling me how enjoyable the conversation was. So, you see how nice it is for both sides.” (I012, professional).

For many, contributing as a peer-expert also created a renewed sense of purpose, especially after periods in which their skills and experiences went unrecognized. Peer-experts felt validated and recognized through their appointment as peer-experts. Several described the role as “An honour! Very nice. Not everyone gets to be involved” (meeting peer-experts C). Their role allowed them to see their personal history as a strength rather than a limitation. The expert-by-experience described how experiential knowledge became a source of credibility rather than something to hide:

“When you've been through so much, at some point you just start to see yourself as very small. And it felt when I turned professional, so to speak, as if that [experience] had to be some kind of hidden part of me, because otherwise I wouldn't be taken seriously. While I increasingly notice that that is precisely why people would choose me. And that gives the feeling of: at least it [referring to her experiences] wasn't in vain.” (I009, expert-by-experience).

Verbal encouragement and visible appreciation played a significant role in reinforcing the sense of value. Peer-experts sometimes needed someone to tell them that they were doing well by a compliment or thank you, as a professional explained: “I think they get a lot of satisfaction from it and do excellent work, but they are not always aware that the good things they are doing can make a difference for others. And they think that’s the most normal thing in the world. That's how it feels a bit.” (I007, professional).

Peer-experts received financial compensation for the work they perform. This is important for both the peer-experts and professionals, as it serves as recognition for their work and it brings joy to have some extra finances. However, some were hesitant to request the available compensation, viewing their contributions as self-evident and not deserving of financial compensation.

Related to the financial compensation, challenges were reported due to organization's complex administrative rules and procedures regarding volunteer remuneration, the maximum legal volunteer compensation, and its potential impact on income-dependent social assistance benefits. The fear of financial consequences caused stress and hesitation for the peer-experts. One professional explained the broader mistrust that underlies this fear:

“So I think that's what it takes for this to be an even greater success. Remove the fear that there will be financial consequences. And of course, the Dutch childcare benefits scandal (The Dutch childcare benefits scandal is a political scandal in the Netherlands involving false allegations of welfare fraud by the Tax and Customs Administration against thousands of families claiming childcare benefits. As a result, they had to repay the entire allowance. Many affected citizens ended up in debt, and some of the victims suffered profound disruption to their lives as a result, including loss of their jobs or homes, mental health problems, and having their children removed from their homes) didn't help either, because those were not just financial consequences. Several people from the neighborhood had their children removed from their homes. Really big consequences. So they are also distrustful of the government. And you cannot say: ‘No, they can be trusted,’ because they see that that is not the case.” (I008, professional).

The appointment as peer-expert was accompanied by mixed feelings for some peer-experts. Some hesitated around the term “peer-expert,” as not all felt comfortable identifying themselves as experts. Therefore, the term “ambassador” was deliberately chosen. While many were proud to be called “ambassadors,” even wearing the accompanying badge with pride, others felt uneasy with the label and worried that it might alienate them from other residents or suggest a hierarchy. Additionally, some peer-experts reported pressure from increased expectations, with fears of making mistakes or “saying the wrong thing” that limited them from openly promoting their role.

#### Cultivating personal growth

Personal growth of the expert-by-experience and peer-experts is being cultivated by an increased level of self-reflection, self-acceptance, and self-confidence.

In general, self-reflection was mentioned as one of the most important skills of experts, particularly the ability to distinguish between their own story and the needs of others. The expert-by-experience emphasized this, stating that the ability to “step outside of your own experience” is essential for offering meaningful support to others (I009, expert-by-experience). Self-reflection of peer-experts is enhanced through the five-day training program peer-experts A and B received, which increased awareness about their own upbringing, parenting style, and interactions with their children. This shifted their views on child development, but also helped them become more conscious and confident in their parenting.

Working as an expert-by-experience or peer-expert also fostered increased self-acceptance. Being seen, heard, and valued for who they are, including their past experiences, helped experts to better accept themselves and shifted their self-perception in a positive direction.

Their role also led to enhanced self-confidence. Being a peer-expert helped them see their own potential and trust their abilities. Many peer-experts are women who received limited formal education, depend on social assistance benefits, have low expectations of themselves, and lack confidence in their abilities. Professionals played an active role in supporting the development of self-confidence by pointing out strengths that peer-experts often overlooked in themselves: “Some already do a lot, but they don’t see that as something important. They do not have that piece of recognition to themselves yet. […] So also that awareness like hey, aren’t you that mother who always stands in the schoolyard and talks with everyone and then refers people to ‘meet’ etc.” (meeting peer-experts C).

For some peer-experts, engaging with the theoretical content of the training was initially intimidating but later empowering. One professional recounted a breakthrough moment: “And during that training one of the peer-experts said: ‘Oh, I didn't think I could do this.’ She suddenly felt an opening to think about training for herself. Because she thought: ‘Oh yes, I can actually do it.’ Yes, that was a really special moment.” (I007, professional).

A challenge can be the varied needs in professional development within the peer-expert role. While many preferred to keep the work and training low-threshold and informal, and free from rigid systems and complex procedures, some voiced a desire for more challenge: “Fantastic mothers who really commit to the neighborhood […] but I notice a difference in what my level is… I want more challenge. I want more homework.” (I014, peer-expert).

#### Acquiring transferable skills and future prospects

Working as a peer-expert contributed to the acquisition of new skills and the development of prospects. Through training and practical experience, peer-experts gained new skills. One peer-expert mentioned that she had significantly improved her Dutch language skills. Others noted how learning about the Dutch school system not only enhanced their ability to support other parents but also helped them navigate educational pathways for their own children.

Additionally, the training and work often prompts participants to reflect on their personal ambitions and to consider new prospects. As a result, several peer-experts began actively pursuing further education or employment. For instance, one peer-expert began a work-study program, another was offered a permanent position at the municipal youth work organization, and a third secured a job at a school with the help of a professional. Professionals view the peer-expert role as a stepping stone for broader life opportunities, particularly for women receiving social assistance benefits:

“Once they are in it, it is very difficult for them to get out of social assistance benefits. And that has to do with several things, including organizing your home situation and your family. So the planning, the organizing, but also how to choose the responsibilities for yourself. […] And if you are such a peer-expert, then you can also practice working together a bit. You can develop some skills that you need in a [professional] job as well.” (I008, professional).

Challenging is that because the role is experienced as meaningful and rewarding, peer-experts can find it difficult to reduce their involvement when personal circumstances, such as financial stress, health issues, or family responsibilities, require it. This highlights the importance of learning to set personal boundaries. Professionals expressed that while guidance and support are essential for skill development and progression, these require time and resources that are not always available. Peer-experts and professionals share the ambition to create more (structural) opportunities for training, education, or employment and envision formalizing the role into a paid function or offering specialized trajectories, while recognizing the varied needs and wishes.

## Discussion

This study identified the impact of being an expert-by-experience or peer-expert within the social domain on the experts themselves. Although literature has described the positive impact that experts-by-experience and peer-experts can have on others regarding health promotion, less is known about how taking on this role affects the experts themselves, particularly in the social domain. We identified four key impacts: feeling connected, feeling valued, cultivating personal growth, and acquiring transferable skills and future prospects.

The expert-by-experience and peer-experts described how the role enhanced their confidence, self-esteem, and sense of meaningful contribution to their communities, consistent with earlier findings (Repper and Carter 2011, Gillard *et al.* 2022, Happell *et al.* 2022). Furthermore, being an expert-by-experience or peer-expert provided new skills, social networks, and educational opportunities that enriched personal development. These impacts have been described as positive work–life spillover effects in other research (Wayne *et al.* 2017, Lapierre *et al.* 2018, MacKenzie 2022). A notable finding regarding working on the basis of experiential knowledge in the social compared with the mental health domain was the feeling of connectedness described by peer-experts. Working together in groups fostered mutual support, recognition, and collective pride, while also enabling reciprocal learning.

Despite these benefits, the role also entails vulnerabilities. Experts-by-experience and peer-experts often face vulnerable or shifting personal circumstances, where increased pressure or relapse remain risks. Particularly in the first months of their work, they may struggle with adjusting to new responsibilities, balancing workload, and managing the emotional intensity of connecting with others (Gillard *et al.* 2022). While challenges in setting boundaries have been noted before (Repper and Carter 2011, Gillard *et al.* 2022, Happell *et al.* 2022), our study highlights that this is particularly difficult for those who both live and work in the same neighborhood. Concerns about financial compensation also emerged as a notable issue. Many peer-experts had a history of financial difficulties and depended on social benefits, making remuneration a sensitive and significant factor to take into account.

In health promotion initiatives that meaningfully integrate experiential knowledge, support from professionals, reciprocal learning in peer groups, and organizational acknowledgment are key mechanisms that foster positive impacts and help prevent negative ones. Supervisors act as crucial sources of support by providing both practical and emotional guidance, mitigating workload, and reorganizing tasks where necessary (Gillard *et al.* 2022, Mirbahaeddin and Chreim 2022, Teborg *et al.* 2024). Reciprocal learning also emerged as essential: people who use their experiential knowledge benefit from working in groups where they support and learn from one another, while their direct interactions with residents also generate new insights that strengthen their own experiential knowledge base and self-growth (Gillard *et al.* 2022). At the organizational level, clear role descriptions, opportunities for development (e.g. training), and an inclusive workplace culture contribute to sustainable engagement and help prevent negative effects (Jacobson *et al.* 2012, Teborg *et al.* 2024). Importantly, not only supervisors but also coworkers shape the work environment: supportive teams and mutual respect create a secure and inclusive setting, while stigma and unmet expectations can have adverse effects (Teborg *et al.* 2024, Schneider *et al.* 2025). Experts-by-experience and peer-experts should be genuinely included, as tokenistic involvement risks undermining their motivation and the value of their role for health promotion (Schneider *et al.* 2025). Finally, sustained development of the expert-by-experience and peer-expert role, recognition of individual differences, and opportunities for career advancement are vital to long-term engagement (Demerouti *et al.* 2001, Schaufeli and Bakker 2004, Gillard *et al.* 2022, MacKenzie 2022).

Finally, this study raises questions about the sustainability and formalization of the expert-by-experience and peer-expert role in health promotion. The role requires considerable investment of time and resources from professionals and organizations. This prompts debate about whether the role should evolve into a protected profession with stable contracts, career pathways, and access to certified education and training. At present, the role and title remain unprotected, not requiring training or education, and inconsistently defined across settings. This flexibility allows for diverse and context-specific contributions but also raises concerns about adequate support, skill development, and role clarity. The Netherlands is currently a frontrunner in this field, being the only country to offer a certified master’s program for experts-by-experience. Expanding suitable training and education opportunities could help experts-by-experience and peer-experts develop essential skills, such as setting boundaries, and enable them to fulfill their bridging function between residents and professionals. It would also prevent tensions with other professionals, such as fears of job substitution (Repper and Carter 2011, Gillard *et al.* 2022). At the same time, peer-experts stressed the importance of keeping the role accessible and low-threshold. A differentiated approach is therefore needed: one that acknowledges the diversity between experts-by-experience and peer-experts offers appropriate education and training for different levels of experiential knowledge and recognizes that working with one’s own lived experience can carry vulnerability, as this study has shown.

Building on these challenges, future research and practice should further explore how to safeguard and strengthen the position of experts-by-experience and peer-experts while preserving flexibility. Firstly, ensuring fair and stable financial arrangements is the key. In our study, monthly payments proved important, and other research has shown that peer workers prefer substantive contracts over casual employment (Gillard *et al.* 2022). Secondly, continued support, clear role descriptions, and structured development opportunities remain crucial. An interesting direction includes mentoring structures in which senior experts guide junior colleagues (Siantz *et al.* 2025). Training tailored to individual needs—such as on boundary-setting and workload management—combined with organizational flexibility can help sustain engagement (MacKenzie 2022, Teborg *et al.* 2024). Thirdly, it is important that supporting experts-by-experience or peer-experts is not a one-size-fits-all process, but rather it requires a tailored approach. This, in turn, places demands on professionals, who may not always have the time or skills to provide such support. Additionally, unique aspects from working on the basis of experiential knowledge in the social domain, such as boundary-setting, sensitivity around financial compensation, and peer-connectedness, should be taken into account. Finally, cross-site collaborations between municipalities and organizations may facilitate implementation and reduce duplication of effort, as sharing experiences and materials enables organizations to build on earlier lessons rather than reinventing the wheel (Schneider *et al.* 2025). Together, these steps may contribute to both professionalizing and diversifying experiential expertise, ensuring its sustainable integration in the social domain and added value to health promotion.

## Strengths and limitations

A key strength of this study lies in its participatory action research design, which fostered trust between researchers and participants and allowed for flexibility to align research methods with participants’ needs. The researchers invested considerable effort in building relationships, which not only enriched the data but also created a safe environment for participants to share their perspectives openly. Using Appreciative Inquiry encouraged participants to reflect on their best experiences and dreams, creating a constructive and forward-looking dialogue about practice (Michael 2005, Cooperrider *et al.* 2008, Beulen *et al.* 2021). This approach stimulated new insights and actions, e.g. initiating conversations with peer-experts about specific challenges and opportunities that were identified. Moreover, knowledge was directly shared with stakeholders interested in applying experiential expertise in other settings.

A limitation of this study is that all peer-experts involved were female, meaning that the findings only reflect experiences from a female perspective. While we did not find literature addressing whether male peer-experts or experts-by-experience experience different impacts or challenges, we cannot exclude the possibility that gender may shape how these roles are experienced, e.g. in relation to emotional expression or boundary-setting. At the same time, the predominance of female peer-experts and experts-by-experience reflects current practice in the social domain, where these roles are more often fulfilled by women. As such, the findings are likely representative of the field, although future research could explore whether and how gender influences experiences of working as a peer-expert or expert-by-experience. Additionally, interviews primarily involved professionals supporting peer-experts. Consequently, the findings may reflect professionals’ interpretations of peer-experts’ experiences rather than peer-experts’ own voices. This limitation was addressed through methodological triangulation, combining interviews and observations to integrate multiple viewpoints and strengthen the robustness of the findings. Participation in group meetings further provided an insider perspective, revealing interactional dynamics that might otherwise have remained hidden, while inclusion of various types of peer-experts enhanced representativeness. Researcher involvement in the data collection may have introduced interpretation bias during data analysis, as the generated codes and themes reflect researchers’ interpretations. Consistent with the principles of reflexive thematic analysis, we treated researcher subjectivity as an integral part of the analytic process by explicitly reflecting on positionality and engaging multiple researchers in coding and interpretation to enhance reflexivity and analytical depth. Additionally, team members who were not involved in data collection reviewed and critically discussed the findings to enhance reflexivity and credibility.

## Conclusion and implications for health promotion

A promising approach to better align health promotion programs with the needs of residents of vulnerable neighborhoods is the engagement of experts-by-experience and peer-experts within the social domain. However, little is known about how taking on this role affects the experts themselves. This study therefore examined the impact of being an expert-by-experience or peer-expert within the social domain on the experts themselves. By fostering connection, recognition, personal growth, and skill development, the roles of peer-experts and experts-by-experience can contribute to empowerment, social inclusion, and improved socioeconomic prospects, which can be regarded as key social determinants of health. At the same time, the identified challenges underscore that the effectiveness and sustainability of peer-based health promotion depends on adequate organizational support. Clear role definitions, mentoring, financial security, and opportunities for development are essential to prevent overburdening and to safeguard the well-being of peer-experts. Social domain–specific positive impacts included peer-connectedness, fostering mutual recognition and a shared sense of purpose, while challenges related to boundary-setting for local peer-experts and sensitivities around financial compensation. Ultimately, health promotion initiatives that meaningfully integrate experiential knowledge and actively leverage the diverse roles of experts, such as mentoring and skill-building, connecting residents to resources, community building, and advocacy, could be better positioned to build trust, address health inequities, and achieve lasting impact in vulnerable neighborhoods.

## Supplementary Material

daag035_Supplementary_Data

## Data Availability

Anonymized data supporting the results in this manuscript are available upon request from the corresponding author.
