# Supplementary material for: How participation in health promotion affects peer-experts and experts-by-experience from vulnerable neighborhoods: a case study from The Netherlands
Source: Health Promot Int. 2026 Mar 18;41(2):daag035. doi: 10.1093/heapro/daag035 (PMC13016922; doi:10.1093/heapro/daag035)
Supplement: daag035_Supplementary_Data [file daag035_supplementary_data.zip › Supplementary File 1. Interviewguide experts-by-experience_clean [003].docx]

**Supplementary File 1. Interview guides for experts-by-experience, peer-experts and professionals**

During the interview, the expert/professional is encouraged to continue talking by asking the following follow-up questions:

- Would you like to explain/elaborate on that further?
- What exactly do you mean by that?
- Can you give an example?
- What happened then?
- How did you come to this conclusion?

**Interview guide for the expert-by-experience and peer-experts**

DISCOVER

Experiences of the expert-by-experience or peer-experts

- What do you like about being an expert-by-experience or peer-expert?
- Can you give an example of a situation in which you experienced your work as very positive, i.e., a situation in which you felt proud, for example?
  - Follow up on an example:
    - Positive for yourself
    - Positive for your family
    - Positive for the residents of the neighborhood
    - Positive for the organization/professionals/project
- (Ask follow-up questions if this has not already been mentioned) What are some things you don't like, find challenging, or difficult about being an expert-by-experience or peer-expert?
- To what extent has being an expert-by-experience or peer-expert changed you in a positive way?
  - Ask follow-up questions about:
    - The effect of any prior training
    - The effect of the work that the expert-by-experience or peer-expert does

Tasks & organization

- How long have you been an expert-by-experience or peer-expert?
- What are your tasks as an expert-by-experience or peer-expert?
- What does your week as an expert-by-experience or peer-expert look like?
- How did you become an expert-by-experience or peer-expert (training, meetings, etc.)?
  - How did you end up in this role?
  - What training or education have you received in the context of your role as expert-by-experience or peer-expert?
  - What have you gained or learned from this?
- Is there any support or are there resources available to you in your work as an expert-by-experience or peer-expert?
  - If so, do you make use of them?
  - Are there things that help?

DREAM

- Imagine it is the year 2030. What would the ideal role of an expert-by-experience or peer-expert look like?
  - What have you achieved as an expert-by-experience or peer-expert?
  - What have you learned?
  - What does the organization/support of expert-by-experience or peer-expert look like?
  - What makes this strategy so ideal?
  - Which elements that you have just described are currently missing?

DESIGN

- What could you do yourself to achieve … [the dreams that have just been mentioned]?
- What is needed from the organization (/professionals) to achieve … [the dreams that have just been mentioned]?
- What is needed from the neighborhood to achieve … [the dreams that have just been mentioned]?
- What is needed in the Netherlands to achieve … [the dreams that have just been mentioned]?

**Interview guide for professionals**

DISCOVER

Experiences of professionals

- What do you like about your job?
- Can you give an example of a situation in which you experienced your work as very positive, i.e., a situation in which you felt proud, for example?
- (If not already mentioned) You also work with peer-experts; can you give an example of a positive experience you had with a peer-expert; i.e., a situation in which you felt admiration/pride/joy, for example?
- (Ask follow-up questions if this has not already been mentioned) What are some things you don't like, find challenging, or difficult about being a professional?

Tasks & organization

- How long have you been working in your current position?
- What are your tasks as a professional with regard to the peer-experts?
  - What does your week look like as a professional?
- Can you tell me why space has been made for experiential expertise in the neighborhood?
  - Why do you consider collaboration with the peer-experts as important?
  - What makes the position of the peer-experts unique compared to other professionals?
- How do residents from the neighborhood become peer-experts? (cues: training, meetings, etc.)
  - How do you think the peer-experts experience this?
- Is there support or are there resources available for the peer-experts?
  - If so, do they make use of them?
  - Are there things that help them?

Being a peer-expert

- Do you think that being a peer-expert has changed the peer-experts in a positive way?
  - Follow up with questions about:
    - The effect of any prior training
    - The effect of the work that the peer-experts do

DREAM

- Imagine it is the year 2030. What would the ideal role of the peer-experts look like?
  - What have you achieved as a professional with regard to the peer-experts?
  - What have the peer-experts achieved?
  - What does the organization/support of peer-experts look like?

DESIGN

- What could you do yourself to achieve … [the dreams that have just been mentioned]?
- What is needed from the peer-experts to achieve … [the dreams that have just been mentioned]?
- What is needed from the organization to achieve … [the dreams that have just been mentioned]?
- What is needed from the neighborhood to achieve … [the dreams that have just been mentioned]?
- What is needed in the Netherlands to achieve … [the dreams that have just been mentioned]?
