# Supplementary material for: How participation in health promotion affects peer-experts and experts-by-experience from vulnerable neighborhoods: a case study from The Netherlands
Source: Health Promot Int. 2026 Mar 18;41(2):daag035. doi: 10.1093/heapro/daag035 (PMC13016922; doi:10.1093/heapro/daag035)
Supplement: daag035_Supplementary_Data [file daag035_supplementary_data.zip › Supplementary File 2. Code list experts-by-experience_clean [002].docx]

**Supplementary File 2. Code list**

**Motivations of the expert-by-experience or peer-experts**

- Desire to help others (high level of engagement, social responsibility and empathy)
- Fairness and equal opportunities
- Social cohesion and mutual respect
- Personal development
- Financial incentives

**The role of the expert-by-experience or peer-experts**

- Mentoring and skill building
  - Setting an example for others: experts demonstrate what they have achieved, thereby offering perspective and validation to others
  - Empowering others: experts identify the strengths of others, acknowledge them, and encourage others to utilize them
  - Translating: translating letters or conversations
  - Emotional support for residents
    - Reassuring others: calming others when they have concerns
    - Support for others: emotional support for others in the form of listening, hugs, and reassurance, among other things
  - Informative support for residents
    - Guiding others: providing parents and their children with information about the transition to school or preschool.
    - Teaching others
    - Providing administrative support to others: helping to fill out forms, order books, etc.
    - Parent-child relationship: informing residents how to deal with (the development of) their child and promoting the relationship with the parent (including advising them to send their child to a school that makes them happy)
    - Helping others connect with Dutch language/culture: explaining how culture and language work in the Netherlands and that sometimes people have to adapt
    - Sparring partner for others: discussing and finding solutions to a particular issue together if the resident cannot resolve it themselves
- Connecting to resources
  - Help from your own pocket or foundation: an expert offers help to residents using their own (material) resources or from a foundation they have set up themselves.
  - Connecting: connecting residents with professionals.
- Community building
  - Organizing something: organizing a support activity for residents.
  - Bringing others along to activities
- Advocacy
  - Speaker with experiential expertise: talking to (large) groups about experiential expertise
  - Contributing ideas for solutions/activities for practice and policy
  - Representative of residents
  - Identifying needs in the neighborhood: experts know what residents need in the neighborhood and communicate this to professionals

**The impact of working as an expert-by-experience or peer-expert**

- Feeling connected
  - Recognition: during their work, experts find recognition in other experts and residents.
  - Support for experts-by-experience or peer-experts
  - Group cohesion
  - Sharing experiences as an expert-by-experience or peer-experts
  - Collaboration between experts-by-experience or peer-experts
  - Frustration
  - Vulnerability
  - Work-life balance: setting boundaries and separating the work of an expert from private life
- Feeling valued
  - Feeling seen and heard
  - Positive energy
  - Pride
  - Sense of purpose
  - Making a difference: feeling that the work experts do makes a difference or can be meaningful
  - Self-acceptance: accepting oneself and one's imperfections
  - Appreciation: appreciation must continue to be expressed to the experts
  - Remuneration
  - Fear of losing social assistance: receiving financial compensation leads to fear of a reduction in social assistance benefits
  - Title of experts-by-experience or peer-experts: disagreement about the title ‘peer-experts’
  - Fear of expectations: experts fear the expectations of others in their role
  - Fear of failure
- Cultivating personal growth
  - Self-acceptance: accepting oneself and one's imperfections
  - Self-reflection: critically examining oneself and one's own beliefs
  - Self-confidence: having or lacking confidence in oneself or self-esteem
  - Self-development: going through a process that has led to self-development (e.g. through training)
  - Perspective on child development: how the child can best develop (mainly that they do not have to do pre-university education if they are happier in vocational education OR, conversely, giving them the opportunity to do higher level education)
  - Support for experts-by-experience or peer-experts
  - Training/education
- Acquiring transferable skills and future prospects
  - Further education/job: education or job as a result of working as an peer-expert
  - Dutch language skills: learning Dutch better
  - Self-development: going through a process that has led to self-development (e.g. through training)
  - Support for experts-by-experience or peer-experts
  - Training/education
  - Work-life balance: setting boundaries and separating the work of an expert from private life
  - Resources for deploying experiential knowledge
